# Internet-delivered CBT intervention (*Space for Sleep*) for insomnia in a routine care setting: Results from an open pilot study

**DOI:** 10.1016/j.invent.2021.100443

**Published:** 2021-08-10

**Authors:** Rebecca Wogan, Angel Enrique, Adedeji Adegoke, Caroline Earley, Sarah Sollesse, Sophie Gale, Marie Chellingsworth, Derek Richards

**Affiliations:** a Clinical Research & Innovation, SilverCloud Health, One Stephen Street Upper, Dublin 8, Ireland; b E-Mental Health Research Group, School of Psychology, Aras an Phiarsaigh, Trinity College Dublin, Dublin 2, Ireland; c Berkshire Healthcare NHS Foundation Trust, Fitzwilliams House, Skimped Hill Lane, Bracknell, England, United Kingdom of Great Britain and Northern Ireland; d The CBT Resource, Exeter, England, United Kingdom of Great Britain and Northern Ireland

**Keywords:** Insomnia, Cognitive behavioral therapy, Digital interventions, Comorbid depression, Comorbid anxiety

## Abstract

Insomnia is a highly prevalent, often comorbid disorder associated with difficulties sleeping, remaining awake, and impaired quality of life. Internet-delivered Cognitive Behavioral Therapy for insomnia (ICBT-I^1^) has the potential to help large numbers of people with sleep disorders. This study investigated the preliminary effects of an 8-week guided ICBT-I intervention within a routine stepped-care service. Fifty-six (N = 56) patients consented to participate. The primary outcome was assessed using the Insomnia Severity Index (ISI) and secondary outcome measures included the Patient Health Questionnaire 9-item (PHQ-9), Generalized Anxiety Disorder 7-item (GAD-7), and the Work and Social Adjustment Scale (WSAS), each administered at baseline and weekly thereafter. Intention-to-treat analyses indicated that ICBT-I produced statistically significant pre- to post- reductions in symptoms of insomnia, yielding within-group effects of *d* = 0.82 suggesting a potential for improved outcomes. Similar improvements were seen across secondary outcomes, with small-to-medium post-treatment within-group effects observed: depression (*d* = 0.63), anxiety (*d* = 0.39), and functional impairment (*d* = 0.31). These findings are supportive of the intervention's potential effectiveness and speak to the importance of several implementation factors that could enhance the effects of the intervention. The results contribute to the growing evidence base for digital interventions designed to help those with sleep difficulties and will inform the design of a future controlled evaluation of ICBT-I under routine clinical settings.

## Highlights

• Evaluated a digital CBT-I program for patients with insomnia in a UK stepped routine care setting for depression and anxiety
• Reductions in symptoms of insomnia post-treatment with a large effect size
• Improvements in comorbid depression, anxiety, and functional impairment outcomes
• Clients experienced rapid and consistent improvements to mental health difficulties
• Usage and patient satisfaction levels were comparable to digital programs for depression and anxiety in the same setting

## 1 Introduction

The annual prevalence for insomnia disorder in the population ranges between 10% and 20%, and approximately 6% of these individuals have a chronic trajectory (Merrigan et al., 2013; Ohayon, 2002). It is characterized by poor sleep quality and/or quantity and may take different forms such as trouble falling asleep, staying asleep, or waking for extended periods, along with difficulties returning to sleep easily, all experienced for at least three months (American Psychiatric Association, 2013; Morin and Benca, 2012). Individuals with the disorder often experience tiredness or sleepiness in the daytime, along with related inattention, irritability and trouble concentrating on tasks, sometimes causing distress that interferes with their work or social life (American Psychiatric Association, 2013). Such complaints increase with age and occur twice as often in women as men (Morin and Benca, 2012). Insomnia also increases the likelihood of developing a mood or anxiety disorder (Li et al., 2016; Mason and Harvey, 2014) and is often exposed as a residual symptom from an episode of depression, and therefore may pose risk for relapse (Manber et al., 2008; Perlis et al., 1997). Chronic insomnia with short sleep duration is related to negative physical health outcomes: including an increased risk of hypertension, diabetes, obesity, heart attack, and stroke (Banks and Dinges, 2007; Knutson et al., 2006; Pearson et al., 2006). In addition to being highly disadvantageous for the individual, there are high societal and healthcare costs relating to insomnia (Riemann et al., 2020).

The most common short-term treatment prescribed for insomnia is pharmacotherapy (Kang and Kim, 2019). However, these medications are often associated with several limitations with long-term use, such as risk of physical dependence, withdrawal, rebound insomnia, and long-term health safety (Buscemi et al., 2007). Psychological interventions, such as Cognitive Behavior Therapy for insomnia (CBT—I), have consistently been shown as effective non-pharmacological alternatives, producing clinically meaningful improvements (Davidson et al., 2019; Van Straten et al., 2017). CBT-I addresses the behaviors, thoughts, and beliefs implicated in the maintenance of the disorder. The intervention can include psychoeducation, relaxation, stimulus control, sleep restriction, and sometimes biofeedback (Perlis et al., 2005). CBT-I is recommended by the American College of Physicians as the first line of treatment for insomnia (Qaseem et al., 2016), and its benefits for persistent insomnia are also acknowledged by the NICE guidelines in the UK (National Institute for Health and Care Excellence, 2004). Indeed, many patients regard CBT-I as more acceptable than medication (Perlis and Smith, 2008; Vincent and Lionberg, 2001). However, in practice, access to these therapies may be restricted through a combination of a lack of trained providers, cost, and a poor understanding of available treatment options (Andrews et al., 2018; Dyas et al., 2010).

One way to increase access to evidence-based treatments for insomnia is to offer CBT-I through the internet, as has been done with interventions for the treatment of emotional disorders including depression and anxiety (Richards et al., 2020; Wright et al., 2019; Zachariae et al., 2016). Recent meta-analyses of studies in this area demonstrate that internet-delivered CBT-I (ICBT-I) can improve insomnia severity, sleep efficiency, subjective sleep quality, wake after sleep onset, sleep onset latency, total sleep time, and number of nocturnal awakenings at post-treatment (Soh et al., 2020; Ye et al., 2015). Results are comparable to those found in similar face-to-face insomnia treatments, with effects maintained into follow-up (Soh et al., 2020; Ye et al., 2015). Furthermore, several studies have shown that ICBT-I also impacts comorbid anxiety and depression in individuals with insomnia (Ye et al., 2015) as well as general health, well-being, and sleep-related quality of life in a general population (Espie et al., 2019).

While there is positive evidence for ICBT-I, few studies have investigated the effects when implemented in routine clinical settings. A recent pragmatic randomized controlled trial (RCT) implementing ICBT-I as an adjunct to routine care for depression in a sample of older men reported moderate to large within-group effect-sizes of the ICBT-I for depressive symptoms and insomnia, respectively, but ultimately could not disentangle the effects of the concurrent interventions (i.e. guideline-based depression care and medication) (Glozier et al., 2019). Similarly, Javaheri et al. (2020) implemented an ICBT-I program within a routine healthcare setting to investigate reductions in insomnia symptoms and improvements to coronary heart disease outcomes, finding preliminary improvements on both insomnia and blood pressure measures. However, these studies conducted in pragmatic routine clinical care settings recruited specialized samples (e.g. older men (Glozier et al., 2019)), patients with coronary heart disease (Javaheri et al., 2020), which limits the generalizability of the results.

The present study attempts to assess the suitability of ICBT-I in the context of the Improving Access to Psychological Therapies (IAPT) service, which is a stepped care approach aiming to increase the availability of treatment for people with depression and anxiety in the UK's National Health System (NHS). The principal objective of this study is to pilot the preliminary effectiveness of the *Space for Sleep* program, an ICBT-I intervention, when used to treat patients with insomnia or reporting sleep difficulties in routine care settings. A secondary aim of this research is to evaluate whether ICBT-I is associated with a reduction in functional impairment as well as comorbid anxiety or depressive symptoms. We anticipate that the current intervention will be associated with preliminary effectiveness in significantly reducing symptoms of insomnia. In line with outcomes from similar digital interventions for depression and anxiety in IAPT (Richards et al., 2020), we expect that symptoms of depression and anxiety will be reduced while functioning scores increase. Further, we assess the trajectory of change in response to ICBT-I over time, facilitated by continuous assessment measures.

## 2 Materials and methods

### 2.1 Trial design

This pilot study followed an uncontrolled design aimed to examine the preliminarily clinical impact of an ICBT-I intervention when offered as part of routine care service delivery. The results of this study will help to establish criteria for the planning of a future large scale RCT study and was conducted following CONSORT guidelines (Schulz et al., 2010) (see Appendix Table A.1). The study was conducted according to the guidelines of the Declaration of Helsinki and the protocol, informed consent, and trial related documents have been approved by the NHS England Research Ethics Committee [Reference 19/YH/0112]. Clinical trials identifier number NCT04493593.

#### 2.1.1 Deviation from trial protocol

We did not report results regarding sleep efficiency data as had been reported in the trial registration page. Sleep efficiency was calculated through the sleep diary entries collected within the intervention, so this information was not gathered before entering the treatment, missing the actual baseline comparison. In addition, few participants entered enough sleep diary entries to compute an average sleep efficiency, leading to low-quality data that was deemed to be unusable to draw conclusions.

### 2.2 Setting

This naturalistic study was conducted within Berkshire Healthcare NHS Foundation Trust Talking Therapies Service, which is an NHS IAPT provider. IAPT has been using SilverCloud as a provider of internet-delivered interventions for some years now. Typically, internet-delivered interventions are offered within the IAPT stepped-care service at Step 2, which provides low intensity therapies suitable for those presenting with mild to moderate symptoms of depression and anxiety. Interventions are delivered to clients and guided by a group of graduate trained clinical staff known as Psychological Wellbeing Practitioners (PWP).

### 2.3 Participants

Inclusion criteria for this study were being aged at least 18 years old and reporting difficulty for at least three months in getting to sleep and/or staying asleep, in line with the diagnosis criteria of the Diagnostic and Statistical Manual of Mental Disorders (DSM-V) for insomnia (American Psychiatric Association, 2013). The IAPT service recognizes that clients may have multiple problems and, rather than administering a single clinical diagnosis, assigns problem descriptors: descriptions of the individual problems participants seek help for considering severity and disability. In this way, a patient may receive multiple problem descriptors if reported difficulties are of equal severity (NHS Digital, 2019). Patients were deemed ineligible for inclusion if they met any of the following criteria: having suicidal intent/ideation, having a psychotic illness, were currently on psychological treatment for sleep disorder, alcohol or drug misuse, having a previous diagnosis of an organic mental health disorder, or having an unstable medication regimen. The decision to end recruitment was made based on time rather than participant numbers, however, a post-hoc power analysis was conducted to assess the statistical power of the trial, see Section 3.2.

### 2.4 Intervention

The program *Space for Sleep* is an internet-delivered CBT-based intervention for insomnia composed of five core modules, plus one unlockable module on sleep restriction (see Table 1). The structure and content of the intervention modules follow evidence-based CBT principles for insomnia (Baglioni et al., 2020). If deemed beneficial for the participant, the PWP can provide access to the unlockable module which otherwise is not visible to users. The unlockable module contains content on sleep restriction of which there are two options: one for regular workers and one for shift workers. The intervention is delivered on a Web 2.0 platform using media rich interactive content. Each module takes roughly 40 min to 1 h to complete and it is recommended that one module be completed per week. Each module is structured to incorporate introductory quizzes, videos, informational content, interactive activities, as well as homework suggestions and summaries. Users are encouraged to keep a sleep diary to monitor and reflect on the impact of these various factors on the current quality of sleep. Participants also complete journal entries, which are open-ended diary-like entries to encourage their own reflective engagement with the intervention content. Journal entries are a tool common to all SilverCloud programs and participants can choose to make the contents viewable to their PWP. The intervention content is delivered in a non-linear fashion, although modules are presented in a recommended order.

**Table 1:** Description of *Space for Sleep* module content.

Module name	Brief description
Getting Started	This opening module provides psychoeducation on sleep, sleep difficulties (e.g. insomnia) as well as the role CBT-I can play in improving their sleep. One of the principle techniques, the sleep diary, encourages users in sleep monitoring and helps them identify important sleep parameters for measuring progress in sleep efficiency throughout the use of the intervention. Psychoeducation on the role of Progressive Muscle Relaxation (PMR) in reducing physical and cognitive tension impacting sleep is also provided, techniques that users are encouraged to practice throughout the intervention.
Good Sleep Habits	This module builds on the previous module and introduces the user to various helping and hindering lifestyle and environmental factors that can impact sleep. The content focuses on improving users' sleep hygiene by creating positive sleeping habits and improving sleep practices (e.g. reduced light exposure and use of technology) for a better bedtime routine.
Improving Sleep Association	This module helps users understand how sleep difficulties may have contributed to a long-term association between bed and having trouble sleeping. It explores underlying reasons and provides stimulus management and control techniques for users in an attempt to make them better acquainted with their own bed/sleep associations (i.e. going to bed only when sleepy, getting out of bed if failing to sleep for 15 min).
Sleeping Less to Sleep Better (Unlockable)	This module introduces a major evidence-based component of CBT-I known as sleep restriction, which provides a set of rules to follow to regulate the sleep-wake schedule by restricting time in bed to only the sleep window (i.e. inducing mild sleep deprivation) to increase the homeostatic drive to sleep that may otherwise have diminished to increase time asleep. Users who disclose that they are employed in shift work can choose to have a version of this module unlocked that is designed specifically for shift workers who require adaptation to the sleep restriction techniques to use them.
Managing Thoughts & Worries	This module helps increase users' awareness of unhelpful thoughts, beliefs, and worries in relation to their sleep difficulties and factors that might be contributing to their poor sleep. Users are provided with cognitive restructuring techniques (e.g. Worry time, Counteract it tool) in an attempt to challenge and change their own unhelpful thoughts and worrying that may exacerbate sleep difficulties. The Counteract it tool encourages the user to think of a competing neutral word or phrase to repeat to oneself, helping to direct thoughts away from any worries or annoyances. The Worry time tool schedules a time each day for purposeful worry, outside of which participants write down any worries to only attend to during worry time.
Keeping Your Sleep Cycle Healthy	This final module, consolidation, helps users to prepare for ending the program by integrating and generalizing the information gained, promoting adherence, helping to identify risk situations, and incorporating strategies to reduce relapse.

### 2.5 Support during treatment

Psychological Wellbeing Practitioners (PWP) are graduate trainees in the provision of low-intensity mental health services in IAPT. As per normal service provision, each participant was assigned a PWP to support and monitor participants' progress through the intervention. Once assigned to the intervention the participant received a message from their PWP, welcoming them to *Space from Sleep*, highlighting aspects of this program, and encouraging them in the use of the program. Over the course of the guided intervention, the PWP logged in every 7–10 days and reviewed participants' progress, leaving feedback for them and responding to the work they had completed, in doing so, spending a recommended 15 min per participant per review. PWPs could view users' goals for the week, tools used, key messages and module satisfaction questionnaires. If users wished to share more, they could share journal entries with their PWP.

### 2.6 Outcomes

#### 2.6.1 Primary outcomes

##### 2.6.1.1 Insomnia Severity Index (ISI)

The 7-item ISI (Bastien, 2001) provides a quantitative index of daytime and night-time impairments of insomnia. Participants rate the severity of sleep problems over the past two weeks (e.g. problems with sleep onset, sleep maintenance, and early morning awakening), interference with daytime functioning, how noticeable the impairment is to others, distress or concern caused by the sleep problem(s), as well as satisfaction with the current sleep pattern. These are rated on a 5-point Likert scale. Scores range from 0 to 28, with higher scores indicating more severe insomnia. Severity categories of the ISI suggest that a score of 0–7 indicates no clinically significant insomnia, 8–14 indicates subthreshold insomnia, 15–21 indicates clinical insomnia with moderate severity, and 22–28 indicates clinical (severe) insomnia. The ISI has been shown to be a valid and reliable measure that is sensitive to changes in treatment studies (Morin et al., 2011; Thorndike et al., 2011).

#### 2.6.2 Secondary outcomes

##### 2.6.2.1 Patient Health Questionnaire-9 (PHQ-9)

The PHQ-9 (Kroenke et al., 2001) is a self-report measure of depression used widely in research and as a screening measure in primary care and hospital settings. The nine items reflect the diagnostic criteria for depression outlined by the DSM-V (American Psychiatric Association, 2013). Summary scores range from 0 to 27, where larger scores reflect a greater severity of depressive symptoms. The PHQ-9 discriminates well between depressed and non-depressed individuals when implementing a cut-off total score ≥ 10 for clinical caseness, showing good sensitivity (88.0%), specificity (88.0%) and reliability (Spitzer et al., 1999).

##### 2.6.2.2 Generalized Anxiety Disorder-7 (GAD-7)

The GAD-7 (Spitzer et al., 2006) comprises seven items measuring symptoms and severity of anxiety. The items are based on the DSM-V diagnostic criteria for GAD (American Psychiatric Association, 2013), which corresponds to a cut-off score of ≥8 for caseness, with higher scores indicating greater severity of symptoms. The GAD-7 has increasingly been used in large-scale studies as a generic measure of change in anxiety symptomatology. The measure has good internal consistency (α = 0.92) and good convergent validity with other anxiety scales (Spitzer et al., 2006).

#### 2.6.3 Work and Social Adjustment Scale (WSAS)

The WSAS (Mundt et al., 2002) is a simple, reliable, and valid self-report measure of impaired functioning, which provides an experiential account of the impact of a disorder. The five questions concern how a disorder impairs the patient's ability to function day-to-day across five dimensions: work, social life, home life, private life and close relationships. Scores range from 0 to 40, with higher score indicating poorer adjustment. WSAS has demonstrated good internal reliability (α = 0.82) and sensitivity to treatment effects (Zahra et al., 2014).

#### 2.6.4 Platform engagement, usage, and acceptability

The online system collects anonymized descriptive data relating to engagement with and usage of the program by the service users. Data collected include the number of modules completed, time spent on the platform, number of activities completed, number of sessions and length of sessions.

Within each module, a module satisfaction questionnaire is completed, consisting of four questions that assess the perceived usefulness and satisfaction with the module. Users are asked to rate “whether they found the module to be (supportive), (relevant), (interesting) and (helpful)” to them. Questions are rated on a 4-point Likert scale to report the user's level of agreement.

### 2.7 Procedure

The *Space for Sleep* ICBT-I intervention has been in use as part of IAPT service evaluation since November 2018. Patients assigned to Step 2 IAPT who were eligible for the ICBT-I intervention were provided with information about the trial and informed that participation would not have any implications on their assigned treatment. As part of IAPT standard procedures, clients attending the service were assessed on their needs, completed the Minimum Dataset (MDS) with a PWP and were assigned a problem descriptor. The MDS is a routine outcome measurement tool used within the IAPT service to enhance care experience, improve service provision, and includes validated outcome measures such as the PHQ-9, GAD-7, and WSAS. As part of this study, they also completed the ISI along with the MDS measures. Participants received an email with information detailing the study and a link to give consent to participate by way of a digital signature. They received information on how to proceed and were informed that they were free to withdraw if they no longer wished to take part.

Throughout the intervention, PWPs scheduled dates with the participants to review their progress within the program. Three days before each review date, as part of IAPT standard procedures, the MDS was triggered and participants were notified to complete the assessments. Thus, the number of assessments completed by the patients was dependent on the number of reviews provided by the PWP.

### 2.8 Statistical methods

To investigate baseline variables, descriptive statistics using *t*-tests and Chi-squared tests were used. Descriptive statistics are reported on platform usage metrics and satisfaction ratings across the modules. Given the naturalistic design of the study, the last available completed MDS was taken as the post-treatment assessment (as per IAPT definition; Clark, 2011). Missing data analysis was conducted to understand the missing pattern in the baseline, post-treatment, and the continuous change scores using Little's ‘missing completely at random’ (MCAR) test and the missing data points were deemed to be MCAR across baseline and post-treatment scores. For the trajectories analyses, the first four subsequent assessments were considered where Time 1 refers to the baseline measure at the start of the participants' treatment, and Time 2 to 4 refer to the successively completed outcome assessments. Timepoint 1 was considered MCAR while for Time 2 to Time 4 data was considered to be ‘missing at random’ (MAR). To handle this missing data, multiple imputation by chained equation via the ‘MICE’ package in R was employed, undertaking five iterations for the imputation process, the imputation method engaged predictive mean matching for the missing data points in the quantitative variables. On occasions, there were more than four assessments completed during the course of treatment. These assessments were not included for analyses due to low completion rates. Specifically, completion rates were at 33.9% (19/56) for the 5th assessment, 17.9% (10/56) for the 6th assessment, and 5.4% (3/56) for the 7th assessment.

Paired sample *t*-tests were used to determine effectiveness of the intervention from baseline to post-treatment across all outcome measures from the estimated data. Effect sizes were calculated as the mean difference from baseline to post-treatment divided by the standard deviation of the difference and interpreted according to Cohen's d benchmarks (Cohen, 1988). Post-hoc power analysis was conducted to investigate the statistical power of the trial based on the sample size, effect sizes for the outcome measures (ISI, PHQ-9, GAD-7 and WSAS), with a two-tailed α of 0.05 Clinically significant change (CSC) was defined as an 8-point reduction in ISI scores from baseline to post-treatment (Morin et al., 2011) and was investigated based on the severity categories of ISI. Categories of recovery and reliable change for PHQ-9 and GAD-7 were defined according to IAPT reporting criteria (National Collaborating Centre for Mental Health, 2020). Standard IAPT reliable change indices (RCIs) were used as cut-offs to measure reliable change on the PHQ-9 (RCI = 6) and GAD-7 (RCI = 4).

For the trajectories of change, linear mixed and marginal models were used to evaluate intervention effectiveness over time, the analyses conducted included all available data from all participants. Statistical checks were conducted to assess model assumptions, and these did not note any major violations. Four models were built across all timepoints, one per outcome variable (ISI, PHQ-9, GAD-7, and WSAS). These models of the trajectories of scores across time were marginal models with an unstructured correlation structure as these produced a superior model fit than corresponding linear mixed models. Bonferroni-adjusted paired comparisons based on estimated marginal means were conducted to explore change between time-points in the intervention. Boxplots of the sample were constructed for the different platform usage metrics (see Section 2.6.2) to spot extreme values: denoted as over 3 times the inter-quartile range. Analyses were conducted in IBM SPSS version 26 and R version 3.6.1 using the ‘lme4’, ‘mice’, ‘mvnmle’, ‘lmerTest’, ‘sjstats’, ‘MuMIn’, ‘BaylorEdPsych’ and ‘emmeans’ packages.

## 3 Results

### 3.1 Baseline characteristics

**Fig. 1:**
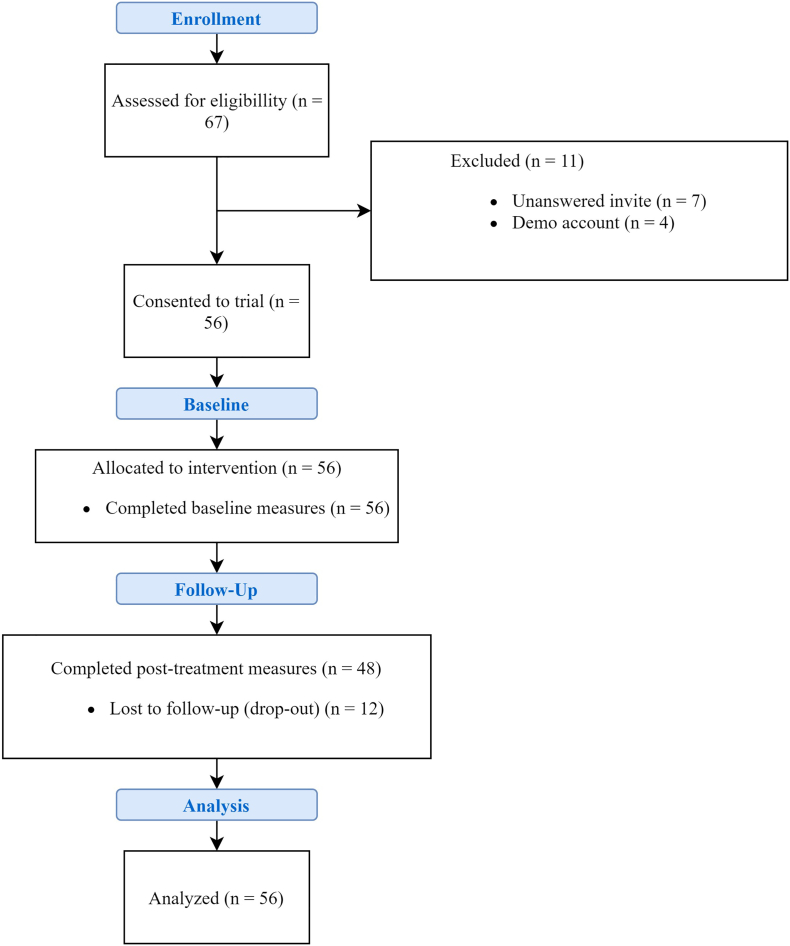
Flow of participants included in the study – CONSORT. Note. Demo account refers to user accounts set up by Psychological Wellbeing Practitioners for training purposes.

Recruitment was conducted over eight months from 15th September 2019 to 12th April 2020 when the last participant began treatment. Of those assessed as eligible for the trial (n = 67), 56 participants provided consent to participate (see CONSORT diagram provided in Fig. 1). The median age of participants was 47 (IQR = 23) and 66.1% were female. At baseline, 66% were employed on a full-time or part-time basis, 8.9% were students, 17.9% were retired or a home-maker, 3.6% were unemployed or not seeking work, and 3.6% had a long-term sickness. In addition to sleep difficulties, IAPT problem descriptors administered to participants were as follows: 52.8% reported a depressive or recurrent depressive disorder, 30.4% reported generalized anxiety, 16.1% reported a somatoform disorder and 1.8% were noted as a non-specified mental disorder.

### 3.2 Effectiveness outcomes

Paired sample *t*-test analyses revealed significant reductions in insomnia (ISI), depression (PHQ-9), anxiety (GAD-7) and functional impairment (WSAS) post-treatment (Table 2). Across the overall sample, a large effect size was found for insomnia severity reduction, a medium effect for depressive symptom reduction, and a small effect for anxiety symptom reduction as well as functioning score improvement as shown in Table 2. Power analyses for the sample on each of the outcome measures (ISI, PHQ-9, GAD-7 and WSAS), with a two-tailed α of 0.05 revealed a power of 100%, 99%, 82% and 61%, respectively.

**Table 2:** Descriptive statistics for pre-post and effect sizes for each of the interventions.

Outcome	Mean		Paired difference Mean (SD)	T-statistics	*p*-Value	Effect size (Cohen's *d*)	95% CI
	Baseline (SD)	Post-treatment (SD)					
ISI	19.1 (5.7)	14.8 (5.9)	4.3 (5.2)	6.2	<0.001	0.82	[0.52, 1.12]
PHQ-9	9.1 (4.4)	6.4(4.7)	2.7 (4.3)	4.7	<0.001	0.63	[0.34, 0.92]
GAD-7	7.1 (4.8)	5.1 (4.7)	2.0 (5.1)	2.9	0.005	0.39	[0.12, 0.66]
WSAS	10.3 (7.0)	7.5 (8.6)	2.8 (9.2)	2.3	0.026	0.31	[0.04, 0.57]

Note. CI, confidence interval; GAD-7, generalized anxiety disorder-7 item questionnaire; ISI, insomnia severity index; PHQ-9, patient health questionnaire-9 item; SD, standard deviation; WSAS, work and social adjustment scale. “Post-treatment” is defined as the last available assessment available in the platform.

Based on the ISI criteria set for clinically significant change (CSC), overall, 27% (15/56) of the sample achieved CSC. When looking at subgroups by baseline severity, 0% (0/2) of those with ‘no clinically significant insomnia’, 11% (1/9) with subthreshold symptoms, 15% (3/20) with moderate symptoms, and 44% (11/25) of those with severe symptoms achieved a CSC by the end of treatment. The rates of reliable change across the secondary outcomes of depression and anxiety were 26.8% (15/56) for the PHQ-9 and 33.9% (19/56) for the GAD-7.

### 3.3 Trajectories of change

**Fig. 2:**
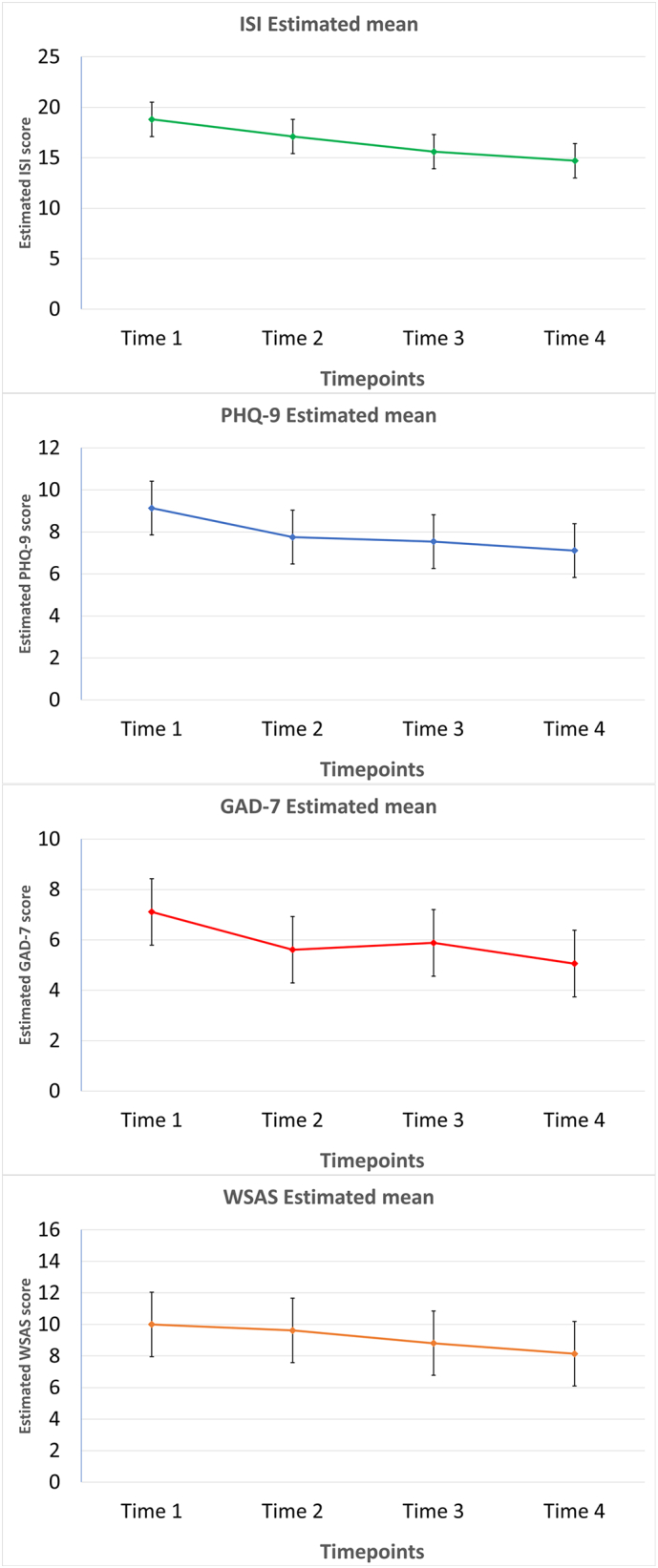
Intervention estimated marginal means across all time-points for ISI, PHQ-9, GAD-7 and WSAS with error bars representing the standard error. Note. Timepoints 1–4 refer to the first four assessments completed during the course of the treatment.

We used linear mixed and marginal models to investigate the evolution of symptoms over the course of treatment where the subjects were included as random effect and time as the fixed effect. Random intercept linear mixed models suggested significant time by subject interaction effects. Paired comparisons confirmed significant improvements from Time 1 to Time 4 on ISI (mean difference = 4.09; SE = 0.74; 95% CI [2.61, 5.57]; *p* < 0.001), PHQ-9 (mean difference = 2.02; SE = 0.52; 95% CI [1.04, 2.99]; *p* < 0.001), GAD-7 (mean difference = 2.05; SE = 0.58; 95% CI [0.89, 3.21]; *p* < 0.01), WSAS (mean difference = 1.86; SE = 0.72; 95% CI [0.42, 3.30]; *p* = 0.06); see Appendix Table A.2 for the model coefficients. Bonferroni estimated mean difference for the participants between Time 1 and Time 4 demonstrated that the models showed improved symptom levels across all timepoints (Fig. 2).

### 3.4 Platform usage

Data relating to engagement and usage of the platform by the service users includes the number of modules completed, time spent on the program, number of activities completed, number of sessions and length of sessions (Table 3). Boxplots revealed cases classified as extreme outliers which were excluded from the analysis in order to prevent sample distribution skewness.

**Table 3:** Program usage across total sample.

Variable	N = 56			
	Mean (min–max)	SD	Median	IQR
Number of logins^a^	15.9 (1–45)	10.0	15.5	13.8
Number of reviews	3.9 (0−12)	2.4	4.0	3.8
Length of use (minutes)^b^	264.4 (7.3–787.7)	186.2	226.8	275.7
Program viewed (%)	56.3 (0–96.9)	29.1	67.7	43.1
Number of activities^c^	35.5 (1−133)	33.7	24.0	37.0

Note. IQR, interquartile range; SD, standard deviation.

a Eight outliers excluded (N = 48).

b Four outliers excluded (N = 52).

c Five outliers excluded (N = 51).

### 3.5 Participant satisfaction

Forty-five (of the 56 participants who consented) completed at least one module satisfaction questionnaire. The total number of questionnaires completed by the 45 participants was 129 over the course of the intervention. Across participant ratings of satisfaction with the eight modules, 92.3% of responses agreed and strongly agreed that the intervention was supportive; 90.7% of responses rated the intervention as relevant; 95.3% agreed and strongly agreed that the intervention was interesting; and 89.9% agreed and strongly agreed that the intervention was helpful, indicating that the majority of participants were satisfied with the *Space for Sleep* program (see Table 4).

**Table 4:** Module ratings across all satisfaction questionnaire responses.

Question	(%) (n)			
	Strongly agree	Agree	Disagree	Strongly disagree
Supportive	25.6% (33/129)	66.7% (86/129)	6.9% (9/129)	0.8% (1/129)
Relevant	31% (40/129)	59.7% (77/129)	7.8% (10/129)	1.5% (2/129)
Interesting	25.6% (33/129)	69.7% (90/129)	3.9% (5/129)	0.8% (1/129)
Helpful	24.8% (32/129)	65.1% (84/129)	9.3% (12/129)	0.8% (1/129)

## 4 Discussion

This pragmatic pilot study aimed to assess the preliminary effectiveness and satisfaction with an ICBT-I intervention (i.e. *Space for Sleep*) offered at step 2 of IAPT and guided by on-site clinical PWP staff. Patients referred to IAPT service who were identified with insomnia as their primary presentation were assigned to the ICBT-I intervention. On average at baseline, participants presented with clinical levels of insomnia with moderate severity and mild symptoms of depression and anxiety. Results showed statistically significant reductions in insomnia severity from baseline to the end of treatment, highlighting the potential for clients to benefit from this intervention. As expected at post-treatment, comorbid symptoms of depression, anxiety, and functional impairment improved significantly with small effect sizes seen for anxiety and functioning improvements and a medium effect size for depression. Participants showed good rates of usage of the platform and reported high satisfaction with the content of the modules.

These results are in line with previous meta-analytic reviews of the efficacy of ICBT-I for individuals with sleep difficulties in research settings (Soh et al., 2020; Ye et al., 2015), which showed positive effects on insomnia severity. However, we did not implement a control group in this feasibility study and so recommend that effect sizes are interpreted with caution due to the within-group nature of the outcome comparisons and before a more robust comparison of outcomes can be conducted. Individual studies of self-guided ICBT-I have previously found within-group effect sizes for improvements in insomnia symptoms ranging from 1.06 (Blom et al., 2015), 1.2 (Lancee et al., 2016) to 2.9 (Van Der Zweerde et al., 2019), which are above the effect size observed in the current study.

Our slightly more moderate effect sizes compared to previously conducted studies may be reflective of the differences between controlled research settings and pragmatic clinical studies conducted in routine care settings (Romijn et al., 2019), which could be due to implementation factors. One of these may be related to how IAPT operates. IAPT case management is geared towards treating depression and anxiety as opposed to insomnia symptoms and it might be the case that patients were discharged on the basis of the reduction of depression and anxiety rather than waiting for a larger reduction in insomnia symptoms. Similarly, perhaps not enough attention was paid to the use of key CBT-I components such as completing the sleep diary entries or the use of the sleep restriction module. Relatedly, the sleep restriction module might have been introduced to participants at a late stage of treatment and perhaps its effects were not captured by the last available measure, given that the effect of this component may take time to unfold. A more long-term follow-up assessment could allow us to both increase benefits for patients but also to aid in capturing a broader picture of the treatment effects. All these factors should be accounted for when training clinicians in the delivery of CBT-I within routine clinical pathways.

Our results suggest that ICBT-I also produces positive effects on comorbid symptoms of depression and anxiety. The improvements to depression were comparable in magnitude to digital interventions aimed specifically at treating depression (Richards and Richardson, 2012); however, the effects on anxiety were much smaller than previously reported for anxiety-focused interventions (Romijn et al., 2019). Cheng et al. (2019) indicated that the risk of developing depression was reduced by half following ICBT-I in a sample recruited from a variety of healthcare settings and the positive effects were maintained at one-year follow-up. As mentioned previously, the relationship between psychological factors and insomnia may be bi-directional (Danielsson et al., 2010; Riemann and Voderholzer, 2003), perhaps with late sleep-onset creating a window for socially-isolated rumination (Kalmbach et al., 2016). In line with previous ICBT-I trials demonstrating co-occurring improvements to psychological well-being, functional health, and quality of life were mediated by improvements to insomnia symptoms (Espie et al., 2019), we also found significant improvements to functioning scores following the intervention. Therefore, the effect of a sleep intervention integrated within a stepped care setting has the potential to act preventatively in reducing symptoms of depression and anxiety before they worsen as well as improving daily functioning but future research should assess this through direct comparisons.

This study examined the trajectories of change in insomnia symptoms over the course of treatment. The evolution of change within treatment can occur immediately or develop with time, while effects themselves may persist or recede (Hollon et al., 2006). The continuous assessment of outcomes revealed relatively linear improvements in insomnia as well as functioning scores. Similarly, Batterham et al. (2017) reported incremental improvements to insomnia symptoms over the course of ICBT-I measuring every two weeks and these were significantly different from baseline measures at every time point including up to 18 months post-treatment. While Morin et al. (2014) showed meaningful improvements to insomnia symptoms following face-to-face CBT gained after two/three weeks of treatment. Our results demonstrated significant improvements across all measurement timepoints indicating a promising trajectory of change in that service users appear to be experiencing rapid and persistent improvements in their difficulties with sleep. A future RCT will be important in investigating whether such promising findings are indeed attributable to ICBT-I by comparing effects across treatment and control conditions. Depression and anxiety scores saw largest reductions from Timepoint 1 to Timepoint 2, with some stagnation at Timepoint 3, before further reductions at Timepoint 4. Again, however, and similar to the initial trajectory mapping for insomnia, a larger sample in any future RCT could allow us to make more temporally detailed models of the trajectories of symptom change but also perhaps to identify subgroups of participants who respond similarly to ICBT-I.

Engagement with online treatment platforms is often determined by both objective and subjective measures which can serve to influence each other (Perski et al., 2017). Objective aspects include the behavioral measures of frequency and duration of use of the platform as well as the use of specific interventional content, for example, tools. Subjective aspects include the focused attention, interest, and enjoyment a user experiences. In this way, engagement varies between and within individuals (Perski et al., 2017). Usage levels of the *Space for Sleep* program were slightly lower but comparable to a previous study using programs for anxiety and depression on the same platform in a similar setting (Richards et al., 2020). These results suggest that under a similar setting and platform, the usage levels are going to remain similar regardless of the program assigned. Subjective ratings of satisfaction with the modules describe the experience of engagement with the program, and overall satisfaction with the modules of the program was rated high. These results combined suggest that the level of engagement with the intervention was appropriate.

### 4.1 Strengths and limitations

This real-world evaluation was conducted within an IAPT stepped-care service which aims to increase the availability of treatments for depression and anxiety in clinical as well as general populations (Clark, 2011). In this study, patients at Step 2 were offered an ICBT-I intervention as their treatment alternative. It is known that insomnia symptoms can be diagnostic of an insomnia disorder or symptomatic of another disorder, e.g. depression or anxiety (Harvey, 2008). Indeed, for some people, seeking help for insomnia may be easier to identify or may involve less stigma than seeking help for anxiety or depression (Glozier et al., 2019). Therefore, this intervention may facilitate treating subclinical comorbidities, while focusing on insomnia symptoms. Likewise, the remote nature of a digital treatment can reduce discomfort in publicly seeking help which may lead to an increased likelihood of treatment adherence, as well as earlier intervention. An additional strength of this study is that we did not exclude shift-workers, a group particularly prone to sleep difficulties, as is often the case in other insomnia treatment interventions (for example, Batterham et al., 2017). Finally, we considered both night and day-related symptoms of sleep difficulties with the use of the ISI, as well as more general health implications such as effects on quality of functioning through use of the WSAS. This may be important to address as it has been proposed that improvement to sleep may mediate the positive effects on depression or anxiety symptoms through improvements to factors such as quality of life, emotion regulation, and cognitive functioning (NHS England, 2019; Palmer and Alfano, 2017).

There were several limitations to this study. Due to convenience sampling within the care setting this study did not calculate an a priori power analysis for the sample size, although retrospective power analyses showed that the main outcomes analyses were well powered to detect differences. We also did not include analyses of sleep efficiency scores, deviating from the trial protocol, as participants' completion of the sleep diaries was too low to calculate a metric over a sufficient number of days. Additionally, the heterogeneity of treatment length due to participants completing treatment at different paces might have resulted in participants receiving different doses of treatment, which may have prevented us from seeing the full treatment effects of the program. The trajectories of change analyses only included the first four assessments and therefore did not capture the continued effects for those patients that completed more assessments. This could have resulted in different results towards the end of treatment and should be further analyzed in future studies. Moreover, implementation of self-report measures of symptoms in this routine care setting, rather than clinical diagnoses, meant that these findings are generalizable to symptoms of sleep difficulties rather than to clinical diagnoses of insomnia. A final limitation concerns the tendency of depression to be more recognizable to mental health professionals than anxiety, even in cases when an individual has depressive episodes stemming from chronic anxiety (NHS England, 2019). Indeed, within the current assessment, over half the participants received a problem descriptor referring to a depressive disorder, while just under a third described anxiety. The IAPT service recognizes that this reported under-detection of anxiety (or over-detection of depression) is an issue and must be addressed by continued assessment of an individual after a problem descriptor of depression has been provided, as an untreated underlying anxiety disorder may lead to recurrence of symptoms (NHS England, 2019).

## 5 Conclusions

This study provides preliminary support for the validity and effectiveness of ICBT-I when offered as the primary treatment alternative in a stepped care setting for the treatment of common mental health disorders. Overall, the observed improvements in insomnia symptoms with concurrent improvements seen for depression and anxiety symptoms highlight the value in offering ICBT-I within a well-established routine care setting. However, our results also suggest room for improvement likely due to factors related to the implementation of ICBT-I within such a routine clinical care setting that will need to be addressed to improve its dissemination and associated outcomes. These findings highlight the value of offering this treatment within the care management pathway of routine mental healthcare, that is, the IAPT stepped care service, and also point to the importance of implementation factors. Indeed, interest in and evidence for ICBT-I is continuing to grow, while digital health interventions and guided self-management in healthcare present opportunities for extensive increases in accessibility and use. Specifically, by addressing sleep problems through the reach of internet-delivered interventions, there is potential for tackling the ever-increasing rates of comorbid depression and anxiety. The future RCT is an important next step in assessing the effectiveness and cost-effectiveness of digital interventions for sleep difficulties, implementing the lessons learned herein, to ultimately provide widespread delivery of ICBT-I as a first-line of treatment within a national health service.

## Funding

This research is funded by SilverCloud Health and Berkshire NHS Foundation Trust.

## Data statement

Patient data presented in this study can be made available upon email request from the corresponding author.

## CRediT authorship contribution statement

Conceptualization, Angel Enrique and Derek Richards; Data curation, Adedeji Adegoke; Formal analysis, Adedeji Adegoke; Funding acquisition, Derek Richards; Methodology, Sarah Sollesse, Sophie Gale and Marie Chellingsworth; Project administration, Caroline Earley; Resources, Sarah Sollesse, Sophie Gale and Marie Chellingsworth; Supervision, Angel Enrique and Caroline Earley; Writing – original draft, Rebecca Wogan; Writing – review & editing, Rebecca Wogan, Angel Enrique, Adedeji Adegoke, Caroline Earley and Derek Richards.

## Declaration of competing interest

Authors Wogan, Enrique, Adegoke, Early, and Richards are employees of SilverCloud Health, developers of computerized psychological interventions for depression, anxiety, stress, and comorbid long-term conditions. SilverCloud Health is a commercial organization that sells its digital programs to commissioners within the NHS who provide the treatment free to patients through the Improving Access to Psychological Therapies (IAPT) service. Authors Sollesse and Gale are employees of Berkshire Healthcare NHS Foundation Trust. Author Chellinsgworth served as a product consultant for the development of the program content.

## Appendix A Supplementary data

Supplementary tables Image 1

## References

[bb0005] American Psychiatric Association (2013). Diagnostic and Statistical Manual of Mental Disorders.

[bb0010] Andrews G., Basu A., Cuijpers P., Craske M.G., McEvoy P., English C.L., Newby J.M. (2018). Computer therapy for the anxiety and depression disorders is effective, acceptable and practical health care: an updated meta-analysis. J. Anxiety Disord..

[bb0015] Baglioni C., Altena E., Bjorvatn B., Blom K., Bothelius K., Devoto A., Espie C.A., Frase L., Gavriloff D., Tuuliki H., Hoflehner A., Högl B., Holzinger B., Järnefelt H., Jernelöv S., Johann A.F., Lombardo C., Nissen C., Palagini L., Riemann D. (2020). The european academy for cognitive behavioural therapy for insomnia: an initiative of the european insomnia network to promote implementation and dissemination of treatment. J. Sleep Res..

[bb0020] Banks S., Dinges D.F. (2007). Behavioral and physiological consequences of sleep restriction. J. Clin. Sleep Med..

[bb0025] Bastien C. (2001). Validation of the insomnia severity index as an outcome measure for insomnia research. Sleep Med..

[bb0030] Batterham P.J., Christensen H., Mackinnon A.J., Gosling J.A., Thorndike F.P., Ritterband L.M., Glozier N., Griffiths K.M. (2017). Trajectories of change and long-term outcomes in a randomised controlled trial of internet-based insomnia treatment to prevent depression. BJPsych Open.

[bb0035] Blom K., Jernelöv S., Kraepelien M., Bergdahl M.O., Jungmarker K., Ankartjärn L., Lindefors N., Kaldo V. (2015). Internet treatment addressing either insomnia or depression, for patients with both diagnoses: a randomized trial. Sleep.

[bb0045] Buscemi N., Vandermeer B., Friesen C., Bialy L., Tubman M., Ospina M., Klassen T.P., Witmans M. (2007). The efficacy and safety of drug treatments for chronic insomnia in adults: a meta-analysis of RCTs. J. Gen. Intern. Med..

[bb0050] Cheng P., Kalmbach D.A., Tallent G., Joseph C.L.M., Espie C.A., Drake C.L. (2019). Depression prevention via digital cognitive behavioral therapy for insomnia: a randomized controlled trial. Sleep.

[bb0055] Clark D.M. (2011). Implementing NICE guidelines for the psychological treatment of depression and anxiety disorders: the IAPT experience. Int. Rev. Psychiatry.

[bb0060] Cohen J. (1988). Statistical Power Analysis for the Behavioral Sciences.

[bb0065] Danielsson N.S., Jansson-Fröjmark M., Linton S.J., Jutengren G., Stattin H. (2010). Neuroticism and sleep-onset: what is the long-term connection?. Personal. Individ. Differ..

[bb0070] Davidson J.R., Dickson C., Han H. (2019). Cognitive behavioural treatment for insomnia in primary care: a systematic review of sleep outcomes. Br. J. Gen. Pract..

[bb0075] Dyas J.V., Apekey T.A., Tilling M., Ørner R., Middleton H., Siriwardena A.N. (2010). Patients’ and clinicians’ experiences of consultations in primary care for sleep problems and insomnia: a focus group study. Br. J. Gen. Pract..

[bb0080] Espie C.A., Emsley R., Kyle S.D., Gordon C., Drake C.L., Siriwardena A.N., Cape J., Ong J.C., Sheaves B., Foster R., Freeman D., Costa-Font J., Marsden A., Luik A.I. (2019). Effect of digital cognitive behavioral therapy for insomnia on health, psychological well-being, and sleep-related quality of life: a randomized clinical trial. JAMA Psychiatry.

[bb0085] Glozier N., Christensen H., Griffiths K.M., Hickie I.B., Naismith S.L., Biddle D., Overland S., Thorndike F., Ritterband L. (2019). Adjunctive internet-delivered cognitive behavioural therapy for insomnia in men with depression: a randomised controlled trial. Aust. N. Z. J. Psychiatry.

[bb0090] Harvey A.G. (2008). Insomnia, psychiatric disorders, and the transdiagnostic perspective. Curr. Dir. Psychol. Sci..

[bb0100] Hollon S.D., Stewart M.O., Strunk D. (2006). Enduring effects for cognitive behavior therapy in the treatment of depression and anxiety. Annu. Rev. Psychol..

[bb0105] Javaheri S., Reid M., Drerup M., Mehra R., Redline S. (2020). Reducing coronary heart disease risk through treatment of insomnia using web-based cognitive behavioral therapy for insomnia: a methodological approach. Behav. Sleep Med..

[bb0110] Kalmbach D.A., Pillai V., Arnedt J.T., Anderson J.R., Drake C.L. (2016). Sleep system sensitization: evidence for changing roles of etiological factors in insomnia. Sleep Med..

[bb0115] Kang S.G., Kim Y.K., Kim Y.-K. (2019). Cognitive behavioral therapy for insomnia in the digital age. Frontiers in Psychiatry. Advances in Experimental Medicine and Biology.

[bb0120] Knutson K.L., Ryden A.M., Mander B.A., Van Cauter E. (2006). Role of sleep duration and quality in the risk and severity of type 2 diabetes mellitus. Arch. Intern. Med..

[bb0125] Kroenke K., Spitzer R.L., Williams J.B.W. (2001). The PHQ-9. J. Gen. Intern. Med..

[bb0130] Lancee J., Van Straten A., Morina N., Kaldo V., Kamphuis J.H. (2016). Guided online or face-to-face cognitive behavioral treatment for insomnia: a randomized wait-list controlled trial. Sleep.

[bb0135] Li L., Wu C., Gan Y., Qu X., Lu Z. (2016). Insomnia and the risk of depression: a meta-analysis of prospective cohort studies. BMC Psychiatry.

[bb0145] Manber R., Edinger J.D., Gress J.L., San Pedro-Salcedo M.G., Kuo T.F., Kalista T. (2008). Cognitive behavioral therapy for insomnia enhances depression outcome in patients with comorbid major depressive disorder and insomnia. Sleep.

[bb0150] Mason E.C., Harvey A.G. (2014). Insomnia before and after treatment for anxiety and depression. J. Affect. Disord..

[bb0155] Merrigan J.M., Buysse D.J., Bird J.C., Livingston E.H. (2013). Insomnia. J. Am. Med. Assoc..

[bb0160] Morin C.M., Benca R. (2012). Chronic insomnia. Lancet.

[bb0165] Morin Charles M., Belleville G., Bélanger L., Ivers H. (2011). The insomnia severity index: psychometric indicators to detect insomnia cases and evaluate treatment response. Sleep.

[bb0170] Morin Charles M., Beaulieu-Bonneau S., Ivers H., Vallières A., Guay B., Savard J., Mérette C. (2014). Speed and trajectory of changes of insomnia symptoms during acute treatment with cognitive-behavioral therapy, singly and combined with medication. Sleep Med..

[bb0175] Mundt J.C., Marks I.M., Shear M.K., Greist J.H. (2002). The work and social adjustment scale: a simple measure of impairment in functioning. Br. J. Psychiatry.

[bb0180] National Collaborating Centre for Mental Health (2020). The improving access to psychological therapies manual. NHS Digital.

[bb0185] National Institute for Health and Care Excellence (2004). Guidance on the use of zaleplon, zolpidem and zopiclone for the short-term management of insomnia. https://www.nice.org.uk/guidance/ta77/resources/guidance-on-the-use-of-zaleplon-zolpidem-and-zopiclone-for-the-shortterm-management-of-insomnia-pdf-2294763557317.

[bb0190] NHS Digital. (2019). Psychological therapies. Additional analyses of therapy-based outcomes in IAPT services (England 2018-19 experimental statistics) (Issue October). https://files.digital.nhs.uk/1C/538E29/psych-ther-2018-19-ann-rep.pdf.

[bb0195] NHS England (2019). The improving access to psychological therapies manual - appendices and helpful resources. NHS Digital.

[bb0200] Ohayon M.M. (2002). Epidemiology of insomnia: what we know and what we still need to learn. Sleep Med. Rev..

[bb0205] Palmer C.A., Alfano C.A. (2017). Sleep and emotion regulation: an organizing, integrative review. Sleep Med. Rev..

[bb0210] Pearson N.J., Johnson L.L., Nahin R.L. (2006). Insomnia, trouble sleeping, and complementary and alternative medicine: analysis of the 2002 National Health Interview Survey data. Arch. Intern. Med..

[bb0215] Perlis M.L., Smith M.T. (2008). How can we make CBT-I and other BSM services widely available?. J. Clin. Sleep Med..

[bb0220] Perlis Michael L., Giles D.E., Buysse D.J., Tu X., Kupfer D.J. (1997). Self-reported sleep disturbance as a prodromal symptom in recurrent depression. J. Affect. Disord..

[bb0225] Perlis M.L., Jungquist C., Smith M.T., Posner D. (2005). Cognitive behavioral treatment of insomnia: a session-by-session guide.

[bb0230] Perski O., Blandford A., West R., Michie S. (2017). Conceptualising engagement with digital behaviour change interventions: a systematic review using principles from critical interpretive synthesis. Transl. Behav. Med..

[bb0235] Qaseem A., Kansagara D., Forciea M.A., Cooke M., Denberg T.D., Barry M.J., Boyd C., Chow R.D., Fitterman N., Harris R.P., Humphrey L.L., Manaker S., McLean R., Mir T.P., Schünemann H.J., Vijan S., Wilt T. (2016). Management of chronic insomnia disorder in adults: a clinical practice guideline from the american college of physicians. Ann. Intern. Med..

[bb0240] Richards D., Richardson T. (2012). Computer-based psychological treatments for depression: a systematic review and meta-analysis. Clin. Psychol. Rev..

[bb0245] Richards D., Enrique A., Eilert N., Franklin M., Palacios J., Duffy D., Earley C., Chapman J., Jell G., Sollesse S., Timulak L. (2020). A pragmatic randomized waitlist-controlled effectiveness and cost-effectiveness trial of digital interventions for depression and anxiety. NPJ Digit. Med..

[bb0250] Riemann D., Voderholzer U. (2003). Primary insomnia: a risk factor to develop depression?. J. Affect. Disord..

[bb0255] Riemann D., Krone L.B., Wulff K., Nissen C. (2020). Sleep, insomnia, and depression. Neuropsychopharmacology.

[bb0260] Romijn G., Batelaan N., Kok R., Koning J., Van Balkom A., Titov N., Riper H. (2019). Internet-delivered cognitive behavioral therapy for anxiety disorders in open community versus clinical service recruitment: meta-analysis. J. Med. Internet Res..

[bb0265] Schulz K.F., Altman D.G., Moher D. (2010). CONSORT 2010 statement: updated guidelines for reporting parallel group randomised trials. BMC Med..

[bb0270] Soh H.L., Ho R.C., Ho C.S., Tam W.W. (2020). Efficacy of digital cognitive behavioural therapy for insomnia: a meta-analysis of randomised controlled trials. Sleep Med..

[bb0275] Spitzer R.L., Kroenke K., Williams J.B.W. (1999). Validation and utility of a self-report version of PRIME-MD: the PHQ primary care study. J. Am. Med. Assoc..

[bb0280] Spitzer R.L., Kroenke K., Williams J.B.W., Löwe B. (2006). A brief measure for assessing generalized anxiety disorder: the GAD-7. Arch. Intern. Med..

[bb0285] Thorndike F.P., Ritterband L.M., Saylor D.K., Magee J.C., Gonder-Frederick L.A., Morin C.M. (2011). Validation of the insomnia severity index as a web-based measure. Behav. Sleep Med..

[bb0290] Van Der Zweerde T., Van Straten A., Effting M., Kyle S.D., Lancee J. (2019). Does online insomnia treatment reduce depressive symptoms? A randomized controlled trial in individuals with both insomnia and depressive symptoms. Psychol. Med..

[bb0300] Van Straten Annemieke, Van Der Zweerde T., Kleiboer A., Cuijpers P., Morin C.M., Lancee J. (2017). Cognitive and behavioral therapies in the treatment of insomnia: a meta-analysis. Sleep Med. Rev..

[bb0310] Vincent N., Lionberg C. (2001). Treatment preference and patient satisfaction in chronic insomnia. Sleep.

[bb0315] Wright J.H., Owen J.J., Richards D., Eells T.D., Richardson T., Brown G.K., Barrett M., Rasku M.A., Polser G., Thase M.E. (2019). Computer-assisted cognitive-behavior therapy for depression. J. Clin. Psychiatry.

[bb0320] Ye Y.Y., Zhang Y.F., Chen J., Liu J., Li X.J., Liu Y.Z., Lang Y., Lin L., Yang X.J., Jiang X.J. (2015). Internet-based cognitive behavioral therapy for insomnia (ICBT-I) improves comorbid anxiety and depression - a meta-analysis of randomized controlled trials. PLoS One.

[bb0325] Zachariae R., Lyby M.S., Ritterband L.M., O’Toole M.S. (2016). Efficacy of internet-delivered cognitive-behavioral therapy for insomnia - a systematic review and meta-analysis of randomized controlled trials. Sleep Med. Rev..

[bb0330] Zahra D., Qureshi A., Henley W., Taylor R., Quinn C., Pooler J., Hardy G., Newbold A., Byng R. (2014). The work and social adjustment scale: reliability, sensitivity and value. Int. J. Psychiatry Clin. Pract..

